# Coffee Pulp Gasification for Syngas Obtention and Methane Production Simulation Using Ni Catalysts Supported on Al_2_O_3_ and ZrO_2_ in a Packed Bed Reactor

**DOI:** 10.3390/molecules28207026

**Published:** 2023-10-11

**Authors:** Carlos Esteban Aristizábal-Alzate, Ana Belén Dongil, Manuel Romero-Sáez

**Affiliations:** 1 Grupo Química Básica, Aplicada y Ambiente—Alquimia, Facultad de Ciencias Exactas y Aplicadas, Instituto Tecnológico Metropolitano—ITM, Medellín 050034, Colombia; manuelromero@itm.edu.co; 2 Instituto de Catálisis y Petroleoquímica, CSIC, Cantoblanco, 28049 Madrid, Spain

**Keywords:** carbon dioxide, methanation, power-to-gas, modeling, biomass valorization

## Abstract

The methanation of CO_2_ is of great interest in power-to-gas systems and contributes to the mitigation of climate change through carbon dioxide capture and the subsequent production of high-added-value products. This study investigated CO_2_ methanation with three Ni catalysts supported on Al_2_O_3_ and ZrO_2_, which were simulated using a mathematical model of a packed bed reactor designed based on their chemical kinetics reported in the literature. The simulated reactive system was fed with syngas obtained from residual coffee pulp obtained after a solvent phytochemical extraction process under several gasification conditions. The results reflect a high degree of influence of the catalyst support, preparation method, and syngas composition on CO_2_ and H_2_ conversions and CH_4_ selectivity. For all the syngas compositions, the Ni/ZrO_2_ catalysts showed the best values for CO_2_ conversion and H_2_ conversion for the Ni/Al_2_O_3_ catalyst except in gasification at 700 °C and using the Ni/ZrO_2_p catalyst.

## 1. Introduction

Nowadays, the methanation of CO_2_, also known as the Sabatier reaction, is of great interest in power-to-gas systems since it produces a substitute natural gas (SNG) and enables the valorization of CO_2_ [1]. Furthermore, the resulting methane is a combustible gas and does not present many difficulties in its storage [1,2]. As CO_2_ is a cause and promoter of climate change, it is highly desirable to develop new processes that capture and/or reuse it [1,2,3] and thus avoid the use of fossil fuels, since their combustion generates more greenhouse gas (GHG) emissions [4,5]. Additionally, being a fuel that can result from the reuse of CO_2_, in principle a zero- or low-carbon footprint can be established once it has been burned, as well as biofuels [6].

Moreover, synthesis gas (syngas) feeds, the raw material used for CO_2_ methanation, can be obtained from the gasification of renewable sources such as agro-industrial residues and municipal organic waste, among others [7,8]. This study investigated syngas obtained from coffee pulp, which is conventionally used as fertilizer, animal feed, and/or low-quality fuel and, if not properly treated, may cause environmental damage [9,10]. Therefore, valorizing coffee pulp promotes green chemical synthesis and follows the principles of a circular economy. It also contributes to obtaining high-added-value products in order to base the economy and consumption patterns on more sustainable activities [11]. However, to incorporate the biorefinery concept, the coffee pulp considered is a byproduct after a phytochemical extraction process with solvents to take advantage of all its potential to generate high-added-value products [8].

Moreover, for the formation of methane from CO_2_, there must be enough H_2_ [3]. Therefore, this study aimed to determine the composition of syngas from coffee pulp gasification using steam as the gasifying agent and varying the steam–biomass ratio (S/B) and operation temperature. Then, it considered simulation of the catalytic conversion of syngas into methane in a packed bed reactor. MATLAB^©^ R2023a software was used to solve the mathematical model. Nickel-based catalysts were extensively studied for this reaction due to their high performance/cost ratio [5,12,13,14], with Ni/Al_2_O_3_ as the most widely used at the industrial scale [1,12,13]. For this reason, in the present work the selected catalyst was based on Ni supported in Al_2_O_3_ and was compared with ZrO_2_ as a support, since this latter oxide shows high activity and selectivity, a high thermal stability, and a low carbon deposition rate in CO_2_ methanation [15,16]. In addition, the considered ZrO_2_ catalysts were prepared using two different methodologies to evaluate the influence of these preparation methods [16]. Based on chemical kinetic models, a commercial Ni/Al_2_O_3_ and two Ni/ZrO_2_ catalysts were compared in terms of CO_2_ and H_2_ conversion and CH_4_ selectivity. The evaluation of the reaction system with all compounds in a packed bed reactor simulation for CO_2_ methanation allowed for a more comprehensive understanding of the process, identification of potential interactions and by-products, and reactor design optimization.

## 2. State of the Art

### 2.1. Syngas from Biomass as a Raw Material for the Methanation Catalytic Process

Gasification is the thermo-chemical conversion of a carbonaceous fuel, and it is characterized as an endothermic process, meaning it requires a heat source [17]. Therefore, it is performed at high temperatures typically ranging from 500 to 1400 °C and can take place under atmospheric or elevated pressures, reaching up to 33 bar [18]. Gasifiers traditionally operate within distinct configurations, including fixed-bed, fluidized-bed, and moving-bed systems [19]. This process involves a partial fuel oxidation by using an oxidizing agent, which could be oxygen, air, steam, or mixtures of these [20,21]. Although using air as a gasifying agent is cheap, the syngas dilution by the N_2_ presence can lead to a reduction in its high heating value as well as a decrease in the overall efficiency of the gasification process [22]. Utilizing steam as the gasifying agent leads to the formation of a syngas characterized by its elevated calorific value, typically ranging from 10 to 15 MJ N/m^3^, and a hydrogen-rich composition [21]. Additionally, Ref. [23] highlights the outcomes of syngas generation through biomass gasification with steam. This work demonstrates a yield enhancing of both hydrogen and carbon dioxide while also presenting a notably higher calorific value in comparison to gasification using oxygen or air as gasifying agents. Therefore, steam was selected as gasifying agent in the present study.

The result of gasification is a fuel gas known as syngas. The main components of syngas are carbon monoxide (CO), hydrogen (H_2_), carbon dioxide (CO_2_), steam (H_2_O_(g)_), methane (CH_4_), nitrogen (N_2_) if the oxidation agent is air or a mixture of it with other agents, some hydrocarbons in a very low quantity, and contaminants such as carbon particles, tar, and ash [17,24]. In the existing literature, the typical syngas composition derived from agro-industrial waste exhibits a hydrogen content falling within the range of 40% to 50%, carbon monoxide at 9.97% to 12.38%, carbon dioxide levels ranging between 25.04% and 26.50%, and consistent methane levels below 0.5% [25]. However, the final syngas composition depends on operational parameters, the type of biomass, and the gasifier configuration [24].

Syngas can be used as a raw material to heat, generate electricity, or synthesize high-added-value chemical and fuel products through several conversion routes, such as methanol or synthetic fuel production [8,11]. Therefore, biomass gasification has been considered a viable option for the conversion/utilization of a variety of feedstocks such as vegetable waste, agro-waste, industrial waste, kitchen waste, food waste, and agricultural waste, and even as the key to a successful substitution for petroleum derivatives [26]. The conditions for this obtention are described in the Materials and Methods section.

### 2.2. Catalytic Methane Production

In CO_2_ methanation, catalysts are needed to achieve high reaction rates, high conversions during CO_2_ hydrogenation, and high selectivity toward methane formation [1,14]. The more reported active metal phases used in this reaction are Ni, Ru, Rh, and Co [1,5,12]. Nickel-based catalysts have been subjected to comprehensive investigation under several reaction conditions due to their relatively low cost and comparatively high catalytic activity [5]. In addition, CO_2_ methanation reaction is affected by the nature of the catalyst support since it plays an important role in the dispersion of metallic sites, CO_2_ adsorption and activation, and metal–support interaction [5,16,27,28]. The most common supports for this reaction are based on metal oxides such as Al_2_O_3_, TiO_2_, SiO_2_, ZrO_2_, and CeO_2_ [5]. Al_2_O_3_- and ZrO_2_-supported nickel catalysts were both reported to be active for CO_2_ methanation, showing a better performance than those that used ZrO_2_ as a support [15,28]. The chemical reactions that can be involved in the methanation process are shown in Equations (1)–(3):

(1) $${\mathrm{CO}}_{2}+4H_{2}\;↔{\mathrm{CH}}_{4}+2H_{2}O$$

(2) $${\mathrm{CO}}_{2}+H_{2}\;↔\mathrm{CO}+{\;H}_{2}O$$

(3) $$\mathrm{CO}+3H_{2}\;↔{\mathrm{CH}}_{4}+{\;H}_{2}O$$

where Equation (1) indicates the chemical reaction of CO_2_ methanation; Equation (2) is the reverse water gas shift (RWGS) reaction; and Equation (3) is CO methanation. The carbon dioxide methanation reaction is exothermic and favored at high pressures and low temperatures [1]. The RWS reaction interferes with the selectivity toward methane production. In theory, CO_2_ methanation is more favorable with an H_2_/CO_2_ ratio equal to or greater than the stoichiometric ratio (4:1) [14].

The catalysts employed in the methanation process are highly susceptible to the presence of impurities in the stream. These impurities can lead to catalyst poisoning and deactivation, thereby diminishing the catalytic performance and overall efficiency [4,29]. The impurities include chlorine and sulfur compounds, ammonia, tars, and particulate matter [29]. The formation of carbon deposits on nickel-based catalysts during the CO methanation reaction has been extensively investigated and can be a significant problem, but it does not pose a concern during the CO_2_ methanation reaction [4]. Therefore, it is better to use a syngas with higher CO_2_ content than CO as a carbon source. Furthermore, the H_2_S presence in the syngas should be analyzed if a real operation is considered.

### 2.3. Ni/ZrO_2_ Catalyst

In the present work, the kinetic information was taken from [16]. In the latter, the two Ni/ZrO_2_ catalysts were tested for methane production by using carbon dioxide hydrogenation. Catalysts were prepared using the incipient wetness impregnation method. According to [16], the impregnated sample was calcinated to obtain NiO/ZrO_2_-C at 500 °C for 3 h, while NiO/ZrO_2_-P was synthesized using dielectric barrier discharge (DBD) plasma for 1 h. Subsequently, the oxidized metal catalysts were subjected to hydrogen reduction at elevated temperatures to form Ni/ZrO_2_-C and Ni/ZrO_2_-P. These catalysts were reduced in situ with pure H_2_ (20 mL/min) at 500 °C for 1 h.

The chemical kinetics proposed in that study did not include a model such as Langmuir–Hinshelwood–Hougen–Watson kinetics, which explicitly considers mass transfer in chemical kinetics. However, simple power-law kinetics for direct reactions can be used for technical applications [30]. According to experimental data reported in [16], the CH_4_ selectivity for both prepared ZrO_2_ catalysts is close to 100%. Therefore, it only considers Equation (1) to develop the kinetic model. Then, the kinetic model of Ni/ZrO_2_ catalysts follows a power-law model as shown in Equation (4).

(4) $$-{r^{\prime }}_{\mathrm{CO}2}=\frac{A}{{\left(R^{\prime }\;*\;T\right)}^{\propto +\beta }}e^{\frac{-\mathrm{Ea}}{R\;*\;T}}{\;*\;P}_{\mathrm{CO}2}^{\propto }{\;*\;P}_{H2}^{\beta }$$

where $-r{’}_{CO2}$ is the kinetic rate for CO_2_; A is a pre-exponential factor related to the chemical kinetics in (L/g·h); R is the ideal constant of gases (8.314 × 10^−3^ kJ/mol); R′ is the ideal constant of gases (0.082 atm·L/K·mol); Ea is the activation energy in kJ/mol; T is the reaction temperature in K, and P_CO2,_ and P_H2_ are the partial pressure of the CO_2_ and H_2_ in atm, respectively. Table 1 shows the values of the chemical kinetic model of the two Ni/ZrO_2_ catalysts.

### 2.4. Commercial Ni/Al_2_O_3_ Catalyst

CO_2_ methanation reaction with a 14–17 wt % Ni/Al_2_O_3_ commercial catalyst was studied in the present work based on the kinetic model established in [1]. The catalyst was reduced under a mixture 50% H_2_ in N_2_ with a total flow of 16 mL/min at 400 °C for 1 h. The main rate expressions for CO_2_ methanation, CO methanation, and RWGS used in the present study are detailed in Equations (5)–(7), respectively.

(5) $${r^{\prime }}_{\mathrm{CO}2\mathrm{meth}}=\frac{k_{\mathrm{CO}2\mathrm{meth}}{\;*\;K}_{H2}{\;*\;K}_{\mathrm{CO}2}{\;*\;P}_{H2}{\;*\;P}_{\mathrm{CO}2}\;*\;\left(1-\frac{P_{\mathrm{CH}4}{\;*\;P}_{H2O}^{2}}{P_{H2}^{2}{\;*\;P}_{\mathrm{CO}2}{\;*\;K}_{\mathrm{eq},\mathrm{CO}2\mathrm{meth}}}\right)}{{\left(1+K_{\mathrm{CO}2}{\;*\;P}_{\mathrm{CO}2}+K_{H2}{\;*\;P}_{H2}+K_{H2O}{\;*\;P}_{H2O}+K_{\mathrm{CO}}{\;*\;P}_{\mathrm{CO}}\right)}^{2}}$$

(6) $${r^{\prime }}_{\mathrm{RWGS}}=\frac{k_{\mathrm{RWGS}}{\;*\;K}_{\mathrm{CO}2}{\;*\;P}_{\mathrm{CO}2}\;*\;\left(1-\frac{P_{\mathrm{CO}}{\;*\;P}_{H2O}}{P_{H2}{\;*\;P}_{\mathrm{CO}2}{\;*\;K}_{\mathrm{eq},\mathrm{RWGS}}}\right)}{\left(1+K_{\mathrm{CO}2}{\;*\;P}_{\mathrm{CO}2}+K_{H2}{\;*\;P}_{H2}+K_{H2O}{\;*\;P}_{H2O}+K_{\mathrm{CO}}{\;*\;P}_{\mathrm{CO}}\right)}$$

(7) $${r^{\prime }}_{\mathrm{COmeth}}=\frac{k_{\mathrm{COmeth}}{\;*\;K}_{H2}{\;*\;K}_{\mathrm{CO}}{\;*\;P}_{H2}{\;*\;P}_{\mathrm{CO}}\;*\;\left(1-\frac{P_{\mathrm{CH}4}{\;*\;P}_{H2O}}{P_{H2}^{3}{\;*\;P}_{\mathrm{CO}}{\;*\;K}_{\mathrm{eq},\mathrm{COmeth}}}\right)}{{\left(1+K_{\mathrm{CO}2}{\;*\;P}_{\mathrm{CO}2}+K_{H2}{\;*\;P}_{H2}+K_{H2O}{\;*\;P}_{H2O}+K_{\mathrm{CO}}{\;*\;P}_{\mathrm{CO}}\right)}^{2}}$$

where $-{r^{\prime }}_{i}$ is the kinetic rate for each reaction; Ea is the activation energy in kJ/mol; Pi is the partial pressure of component i in bar; Ki is the adsorption constant of component i; and Keq, i is the chemical equilibrium constant for reaction i. Table 2 and Table 3 show the adsorption and kinetic parameters, respectively, of this catalyst model.

## 3. Results and Discussion

### 3.1. Biomass Chemical Composition

The chemical composition of the residual coffee pulp was expressed by its elemental analysis. Table 4 shows the C, N, H, O, and S content in the sample on a dry basis. 

The coffee pulp showed a low hydrogen content, which recommended using a gasifying agent, such as steam, capable of supplying an increment to the syngas H_2_ concentration [31]. Furthermore, the sulfur content was very low or negligible. However, the obtained syngas showed low quantities of H_2_S, which indicated that in the elemental analysis, the sulfur was below the detection limit. Therefore, it is possible the sulfur content was below the limit detection. Elemental analysis of the coffee pulp showed a similar CHNO ratio than other residual biomasses reported in [32]. This study suggests that the elemental analysis composition is attributed to presence of lignin and cellulose in the biomass, which is a typical feature of agro-industrial waste.

### 3.2. Syngas Description

We performed the gasification of the coffee pulp at two temperatures and two steam/biomass (S/B) ratios. The compositions of the obtained syngas in %*v*/*v* are shown in Table 5. S/B ratios were used because steam was the gasifying agent selected in this work. Assuming an ideal gas behavior for syngas, the composition in %*v*/*v* is equivalent to the molar fraction.

According to the results described in Table 5, the formation of CO_2_ was favored under conditions where the S/B ratio was 0.5 and 1.0 at 700 °C. However, there was no significant variation in temperature that allowed establishing a better operating condition for this gasification parameter. However, at a gasification temperature of 700 °C, a slightly higher amount of CO_2_ was achieved for an S/B ratio of 0.5. On the other hand, higher amounts of H_2_ were achieved, as for CO_2_, for both S/B ratios at 700 °C. Additionally, upon analyzing the considered reactions, CO could contribute to methane formation according to reaction B. Therefore, according to the data in Table 5, better production was achieved for both S/B ratios and a temperature of 800 °C. However, at this temperature, the production of CO_2_ was not high as at 700 °C. The H_2_/CO_2_ ratios were greater than the stochiometric ratio for reaction 1 (4:1) when a gasification temperature of 800 °C for both S/B ratios was implemented. Furthermore, with an S/B of 0.5, a better value for this relation was achieved.

According to [21], raising the gasification temperature leads to a reduction of chemical species that can poison and enhance catalyst deactivation. However, this was true for our own gasification results when an S/B of 1.0 was used, because at an S/B ratio of 0.5, the behavior was contrary to what was established by these references. Therefore, the S/B ratio could affect the formation of H_2_S since there was a clear variation in the formation of this poison agent when comparing both gasification temperatures. To obtain synthesis gases from coffee pulp with the most adequate composition to avoid the possible acceleration of catalyst poisoning and deactivation, a temperature of 800 °C and an S/B of 1.0 should be selected because they presented the lowest value for H_2_S formation in the syngas. 

### 3.3. Catalytic Simulation of Methanation 

**Figure 1:**
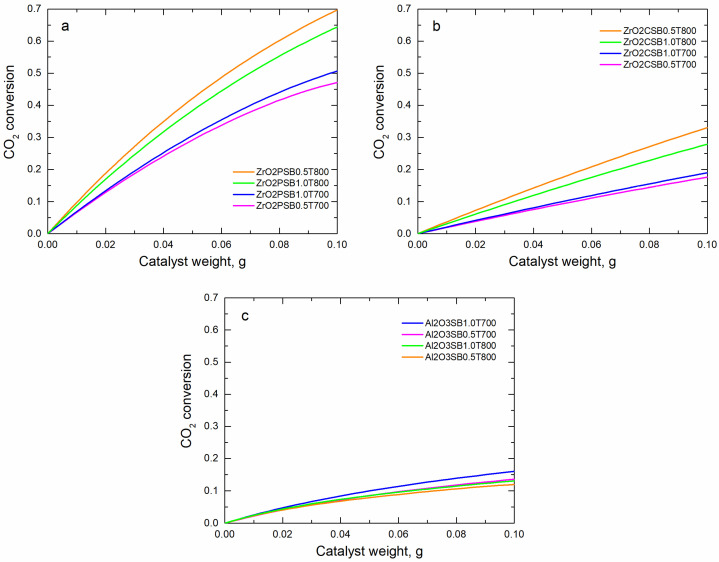
CO_2_ conversion of Ni-based catalysts for all syngas compositions: (**a**) Ni/ZrO_2_-P; (**b**) Ni/ZrO_2_-C; (**c**) commercial Al_2_O_3_.

**Figure 2:**
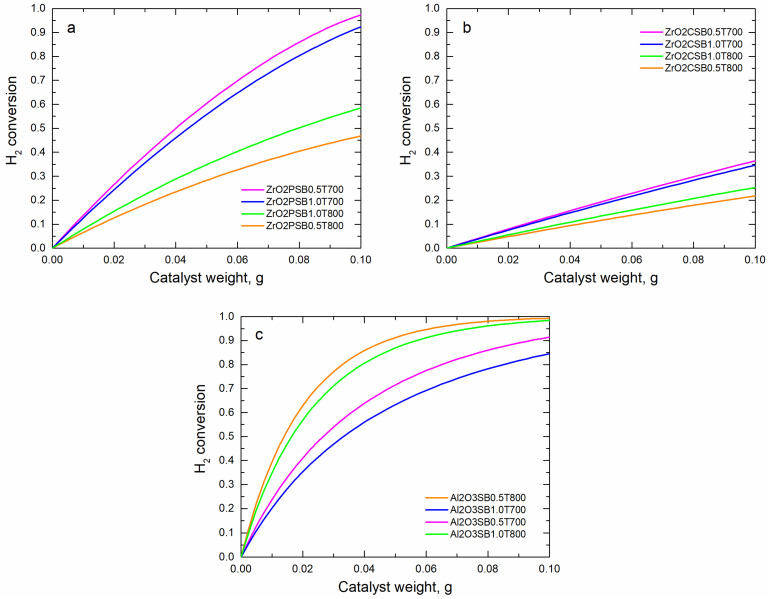
H_2_ conversion of Ni-based catalysts for all syngas compositions: (**a**) Ni/ZrO_2_-P; (**b**) Ni/ZrO_2_-C; (**c**) commercial Al_2_O_3_.

Once we obtained the syngas compositions after gasification, we analyzed the CO_2_ conversion rate and H_2_ conversion throughout the weight catalytic bed (w) of each one of the syngas compositions and catalysts while considering the kinetics described above. The results are shown in Figure 1 and Figure 2. The nomenclature used in all figures with a legend contain the catalyst type followed by the S/B ratio and gasification temperature.

At 800 °C gasification for both S/B ratios, the feed syngas streams contained a lower concentration of CO_2_, resulting in a decreased reactant-to-catalyst ratio; in other words, it had a lower space velocity. This condition led to higher conversion rates for these gasification process parameters. To obtain an accurate comparison, it was necessary to evaluate the results obtained at 700 °C and those obtained at 800 °C separately for all simulated catalysts. As shown in Figure 1 and Figure 2, Ni/ZrO_2_-P obtained better results than Ni/ZrO_2_-C in terms of CO_2_ conversion and H_2_ conversion. In accordance with [16], this behavior was due to a greater number of active sites, which could be provided by highly dispersed Ni particles on Ni/ZrO_2_-P, leading to a higher reaction rate for CO_2_ methanation. The surface Ni concentration of Ni/ZrO_2_-P, estimated at Ni/Zr = 0.18, was higher than that for the Ni/ZrO_2_-C catalyst (Ni/Zr = 0.13). Therefore, this indicated a better Ni dispersion on the support induced by plasma decomposition. In addition, it has been proposed that this method of preparation facilitates the partial reduction of ZrO_2_ to create more oxygen vacancies, thus improving CO_2_ activation [16]. For these ZrO_2_ supported catalysts, at 700 °C it is better to use an S/B ratio of 1.0 to obtain high conversions for CO_2_. However, for the same temperature, the H_2_ conversion is best when the Ni/ZrO_2_-C catalyst uses a syngas for an S/B of 1.0 and for Ni/ZrO_2_-P, an S/B of 0.5. The highest H_2_ conversion was produced at 800 °C with an S/B ratio of 1.0 and CO_2_ conversion at an S/B of 0.5. 

Regarding the results for the commercial Ni/Al_2_O_3_ catalyst in Figure 1c, it achieved the highest value of CO_2_ conversion with a syngas obtained using an S/B ratio of 1.0 for both temperatures in the gasification process. According to Figure 2c, the H_2_ conversion behavior of this support was opposite for the mentioned S/B gasification condition. 

**Figure 3:**
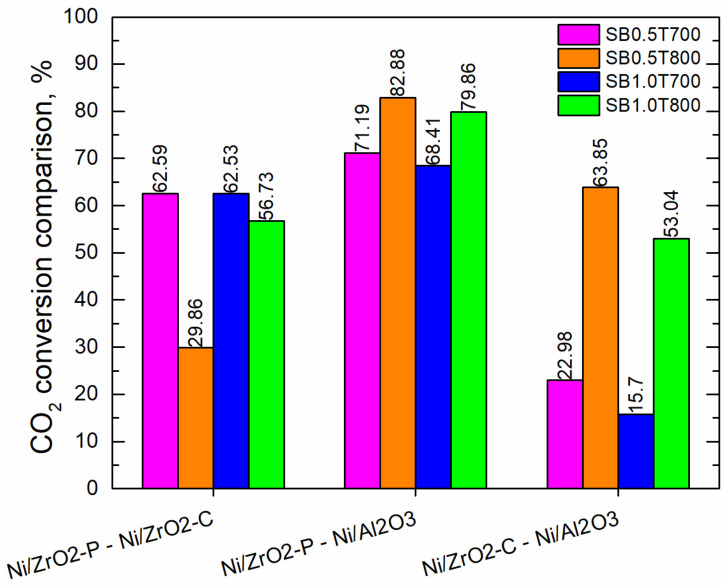
Comparison in percentage (%) of CO_2_ conversion for the catalysts under study.

**Figure 4:**
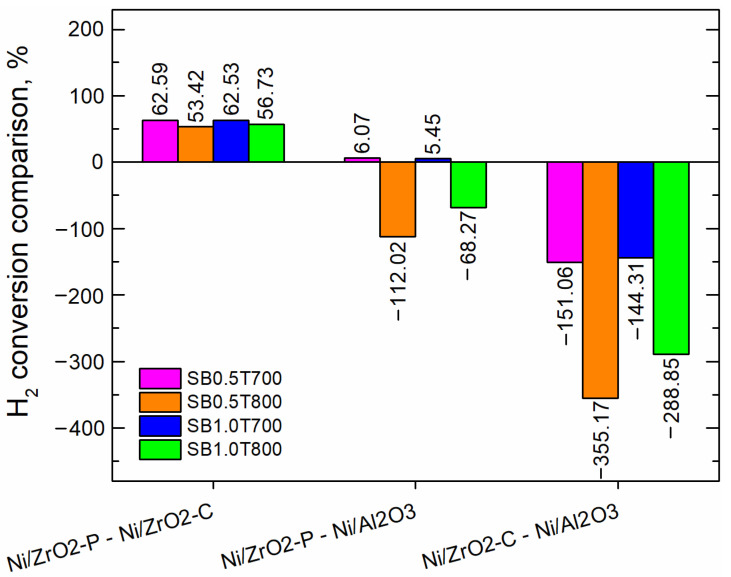
Comparison in percentage (%) of H_2_ conversion for the catalysts under study.

Figure 3 and Figure 4 show the comparisons of CO_2_ and H_2_ conversions, respectively, obtained with the different catalysts at the reactor outlet using all the syngas compositions. A positive and negative value indicate a stronger influence of the variable in the first and second position in the comparison, respectively. For instance, in the comparison between ZrO_2_p and Al_2_O_3_, positive values indicate a higher conversion of ZrO_2_p, while negative values indicate a greater conversion of Al_2_O_3_.

Figure 3 shows that the highest values of CO_2_ conversion were achieved with ZrO_2_. This result is in accordance with [15], which reported that the catalytic system exhibited a higher activity with ZrO_2_ supports than with Al_2_O_3_. ZrO_2_p shows better conversions than ZrO_2_c for all syngas compositions. Additionally, it could be attributed to a pre-exponential factor, and the reaction order was greater for Ni/ZrO_2_p than for Ni/ZrO_2_c. Therefore, the CO_2_ methanation must be faster for the Ni/ZrO_2_p catalyst. On the other hand, Figure 4 shows that H_2_ conversion had the most prevalent negative values for Ni/ZrO_2C_ compared to Ni/Al_2_O_3_. 

**Figure 5:**
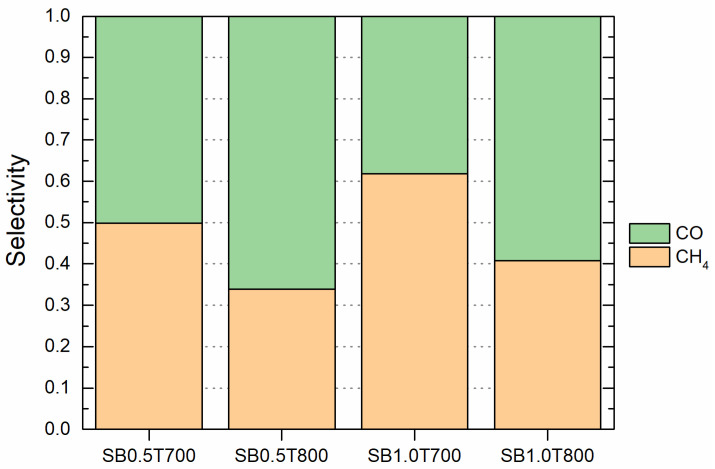
Selectivity for commercial Ni/Al_2_O_3_ catalyst considering all the gasification conditions (S.B0.5T700 means a syngas produced using an S/B ratio of 0.5 and a gasification temperature of 700 °C).

Since the power-law kinetic model for the Ni/ZrO_2_ catalysts only included Equation (1) for CO_2_ methanation, it was not possible to establish the CO selectivity. However, chemical kinetic model for the commercial Ni/Al_2_O_3_ catalyst considered all reactions. Therefore, this catalyst allowed us to calculate the selectivity for CO, to determine the performance of gasification conditions in Sabatier process to increase the CH_4_ yield, and to minimize the by-product generation. Figure 5 illustrates the CH_4_ and CO selectivity for the commercial Ni/Al_2_O_3_ catalyst when considering all the gasification conditions.

Figure 5 shows a better behavior of the Ni/Al_2_O_3_ catalyst for CO selectivity at 800 °C when an S/B of 0.5 was applied. At 700 °C, the same effect is shown for an S/B of 1.0. Furthermore, with these gasifying conditions, the CH_4_ selectivity was lower. Despite achieving an H_2_/CO_2_ ratio of less than 4 under these gasification conditions, the concentration of CO_2_ was higher than that of CO. This predominance of CO_2_ concentration plays a crucial role in methane (CH_4_) production. According to the adsorption parameters of the Al_2_O_3_ catalyst given in Table 2, it was observed that CO_2_ adsorption was stronger compared to CO. However, even though the kinetic constant for CO methanation was greater and the activation energy was lower than that for CO_2_ methanation, this result implies that the rate-limiting step in methane production using the Ni/Al_2_O_3_ catalyst was the adsorption of reactants. According to the results, a stronger influence of the S/B ratio was observed for CH_4_ selectivity for both employed gasification temperatures. 

## 4. Materials and Methods

This section starts with a detailed description of the biomass elemental analysis and gasification procedure for syngas production from coffee pulp after a solvent phytochemical extraction to obtain chlorogenic acid. These residual coffee pulps were taken as a solid filtered after the extraction process using ethanol/H_2_O (70/30 %*v*/*v*) at ambient temperature, atmospheric pressure, and a coffee pulp-to-solvent ratio of 1 to 4. Then, a theorical kinetic study is considered for methane production using Ni-based catalysts on ZrO_2_ and Al_2_O_3_ supports on a packed bed reactor. It presents a mathematical model that includes the assumptions and all the equations that are necessary to simulate a packed bed reactor considering a heterogenous catalyst for the reactive system. Finally, it details the CO_2_ conversion, H_2_ conversion, and CH_4_ selectivity of the model constructed here when it was simulated with each catalyst using its respective chemical kinetic parameters found in the cited papers.

### 4.1. Elemental Analysis

The elemental analysis of the coffee pulp sample was determined using a LECO CHNS analyzer (Truspec micro model). The procedure was developed following the ASTM D-5373-08 method. The CHN analysis was conducted at 1050 °C and the sulfur analysis at 1350 °C, both in a helium atmosphere. The results are reported on a dry basis. Since the sulfur content was negligible, the oxygen content was calculated according to the difference [32].

### 4.2. Gasification Experimental System

**Scheme 1:**
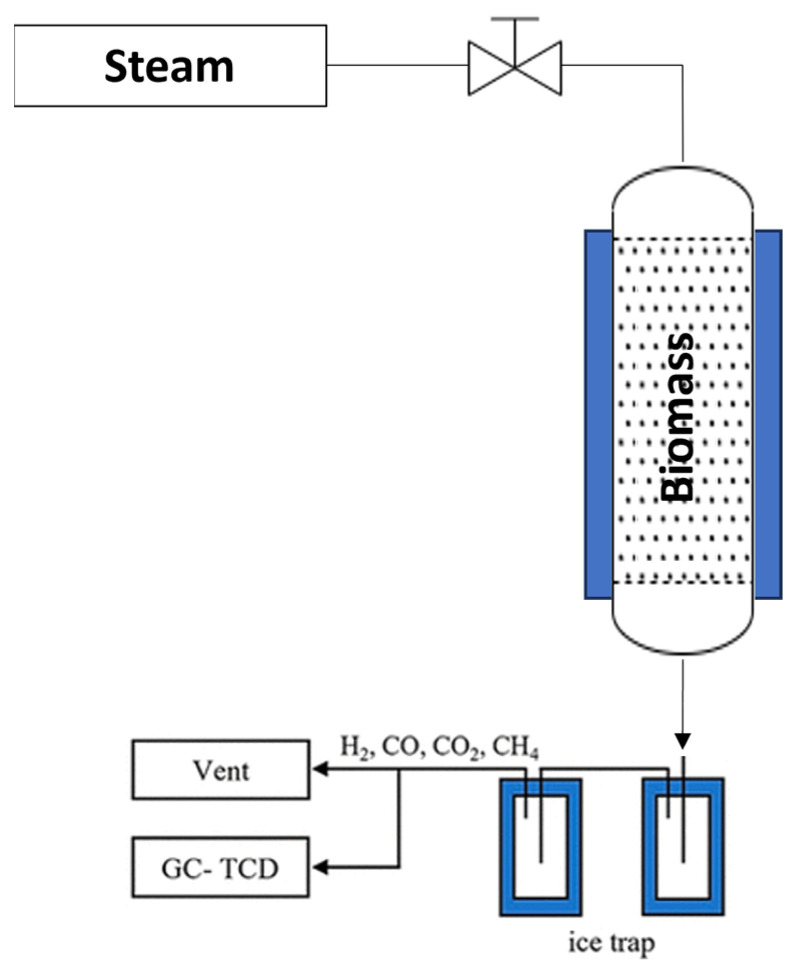
Fixed bed gasifier with external furnace.

The residual coffee pulp was subjected to gasification using a horizontal furnace reactor. The reactor comprised a quartz tube with an external diameter of 3.5 cm (internal diameter of 3.0 cm) and a length of 50 cm. The quartz tube was inserted into the annular space of the 40 cm long horizontal furnace. The furnace was heated by electric resistances, and its temperature was controlled through a proportional–integral–derivative (PID) loop, enabling continuous monitoring of the reactor temperature. To facilitate the gasification process, the sample was loaded into a quartz sample holder that was 2.0 cm in diameter and 13.5 cm in length within the reactor. Inside the sample holder, a K-type thermocouple was inserted to accurately measure the temperature of the sample bed. Scheme 1 shows a gasifier diagram employed in this study.

The gasification experiments were conducted at two different temperatures: 700 °C and 800 °C. Additionally, two steam-to-biomass (S/B) ratios were tested, namely 0.5 and 1.0, to evaluate their effects on the gasification process. For each S/B ratio and temperature, seven experiments were conducted with variations in the gasification time. The first sample was analyzed at 6 min of the gasification process, the second was at 10 min, and subsequent analyses were conducted every 10 min until reaching 60 min of gasification. After that, the arithmetic average of these seven measurements was taken as the result for the syngas composition.

The produced syngas was analyzed using an Agilent Micro GC model 3000. This analytical instrument was equipped with two thermal conductivity detectors (TCD). One TCD utilized a 10 m × 0.32 mm 5A molecular sieve column with argon as the carrier gas, while the other TCD employed an 8 m × 0.32 mm column with helium as the carrier gas.

### 4.3. Mathematical Model for the Methanation Catalytic Packed Bed Reactor Simulation

This subsection discusses the simulation of a packed bed reactor operating in steady-state conditions. In the simulation, the reactor considered a catalyst weight of 100 mg in a packed bed configuration, 1 atm of pressure, a total inlet molar flux of 0.1 mol/min, ambient pressure, and 400 °C. This temperature was selected since in previous studies, at the optimal temperature range of 350 to 450 °C, Ni/Al_2_O_3_ catalyst systems synthesized through conventional methods typically exhibited CO_2_ conversion rates ranging from 50% to 80% and CH_4_ selectivity exceeding 90% [14]. Another reason to choose this temperature was because the reaction rate is relatively slow at low temperatures. However, CH_4_ selectivity is higher at these operating temperatures [15]. Therefore, these conditions were maintained constant in all the simulations to be able to compare the behavior of the catalysts using the syngas compositions described above. The following considerations and simplifications proposed in [33,34] were implemented for this type of operation and reactor:

- Negligible radial diffusion: concentration and temperature profiles were assumed to be constants, which led to a one-dimensional model.
- Constant radial speed.
- Temperature and pressure profiles in the catalyst were assumed to be constants (homogeneous catalytic particles).
- As in [13], the mechanisms related to catalyst deactivation, such as sulfur poisoning or carbon formation via the Boudouard reaction, were not taken into consideration or disregarded in the present study.

As the process was carried out in a packed bed reactor, the resulting model had to be adjusted to the design equation of this type of reactor, which can be consulted in [13]. Equations (8)–(12) are the system of ordinary differential equations (ODEs) used here for the mass balance and reactor design. They express each reactive species involved in the Sabatier process.

(8) $$\frac{{\mathrm{dF}}_{\mathrm{CO}}}{\mathrm{dW}}={r^{\prime }}_{\mathrm{CO}}$$

(9) $$\frac{{\mathrm{dF}}_{\mathrm{CO}2}}{\mathrm{dW}}={r^{\prime }}_{\mathrm{CO}2}$$

(10) $$\frac{{\mathrm{dF}}_{H2}}{\mathrm{dW}}={r^{\prime }}_{H2}$$

(11) $$\frac{{\mathrm{dF}}_{\mathrm{CH}4}}{\mathrm{dW}}={r^{\prime }}_{\mathrm{CH}4}$$

(12) $$\frac{{\mathrm{dF}}_{H2O}}{\mathrm{dW}}={r^{\prime }}_{H2O}$$

where Fi denotes the molar flux of species i; r′i is the chemical kinetics of species i; and W is the catalyst weight inside the heterogeneous reactor in grams.

Since the system had three chemical reactions in parallel; Equation (1) CO_2_ methanation, Equation (2) RWGS, and Equation (3) CO methanation), it needed to consider the species that appeared in more than one of these reactions because the chemical kinetics of each of the said reactions were coupled and given in parallel as well. Equations (13)–(17) show the global or total reactions for each species involved in the process described above, where each r_i_ is expressed in mol/min·g_cat_.

(13) $${r^{\prime }}_{\mathrm{CO}2}=-({r^{\prime }}_{\mathrm{CO}2-\mathrm{met}}+{r^{\prime }}_{\mathrm{RWGS}})$$

(14) $${r^{\prime }}_{H2}=-(4.{\;r^{\prime }}_{\mathrm{CO}2-\mathrm{met}}+{r^{\prime }}_{\mathrm{RWGS}}+3.{r^{\prime }}_{\mathrm{CO}-\mathrm{met}})$$

(15) $${r^{\prime }}_{\mathrm{CH}4}={\;r^{\prime }}_{\mathrm{CO}2-\mathrm{met}}+{r^{\prime }}_{\mathrm{CO}-\mathrm{met}}$$

(16) $${r^{\prime }}_{H2O}=2.{r^{\prime }}_{\mathrm{CO}2-\mathrm{met}}+{r^{\prime }}_{\mathrm{RWGS}}+{r^{\prime }}_{\mathrm{CO}-\mathrm{met}}$$

(17) $${r^{\prime }}_{\mathrm{CO}}={r^{\prime }}_{\mathrm{RWGS}}-{r^{\prime }}_{\mathrm{CO}-\mathrm{met}}$$

Catalyst performance was analyzed here by measuring the CO_2_ and H_2_ conversion and the selectivity of CH_4_ and CO. CO_2_ and H_2_ conversion are described in Equations (18) and (19), and the selectivity of CO to CH_4_ are described in Equations (20) and (21).

(18) $$X_{\mathrm{CO}2}=\frac{F_{\mathrm{CO}2_{\mathrm{in}}}-F_{\mathrm{CO}2_{\mathrm{out}}}}{F_{\mathrm{CO}2_{\mathrm{in}}}}$$

(19) $$X_{H2}=\frac{F_{H2_{\mathrm{in}}}-F_{H2_{\mathrm{out}}}}{F_{H2_{\mathrm{in}}}}$$

(20) $$S_{\mathrm{CO}2/\mathrm{CH}4}=\frac{F_{\mathrm{CH}4_{\mathrm{out}}}}{F_{{\mathrm{CO}}_{\mathrm{out}}}+F_{\mathrm{CH}4_{\mathrm{out}}}}$$

(21) $$S_{\mathrm{CO}2/\mathrm{CO}}=\frac{F_{{\mathrm{CO}}_{\mathrm{out}}}}{F_{{\mathrm{CO}}_{\mathrm{out}}}+F_{\mathrm{CH}4_{\mathrm{out}}}}$$

where X_CO2_ and X_H2_ are the CO_2_ and H_2_ conversion inside the reactor and S_CO2/CH4_ and S_CO2/CO_ are the selectivity of CO_2_ to transform it into CH_4_ and CO, respectively; and F_i_in_ and F_i_out_ are the molar flux of components according to the subindex in mol/min.

Most of the kinetic expressions studied and analyzed here depend on the partial pressures of the chemical species involved, and the system of differential equations was based on molar balance. Therefore, Equation (22) must be used to relate the partial pressures to the molar fluxes of each species.

(22) $$P_{i}=\frac{F_{i}}{\sum_{i=1}^{n}F_{i}}{\;*\;P}_{T}$$

where P_i_ is the partial pressure of species i inside the reactor; F_i_ is the molar flux of component i (mol/min); $\overset{n}{\underset{i=1}{\sum }}F_{i}$ is the summation of all molar fluxes of the species involved (mol/min); and P_T_ is the total system pressure.

## 5. Conclusions

This paper presented a coffee pulp gasification after a phytochemical extraction process with varying S/B ratios (0.5 and 1.0) and temperatures (700 °C and 800 °C). The resulting syngas was considered as a raw material from methane production through the Sabatier process. The adequate syngas according to the H_2_/CO_2_ ratio was achieved at 800 °C and both S/B ratios because these conditions exceeded the stoichiometric ratio (4:1) for the CO_2_ methanation reaction. Additionally, the temperature of 800 °C reduced the formation of H_2_S, which favored avoiding the acceleration of catalyst poisoning. However, in the present study, this effect was not considered in the catalyst simulation.

Then, a comparative analysis of three Ni catalysts in terms of CO_2_ methanation performance and behavior was considered. Such analysis was based on a mathematical model that simulated their chemical kinetics reported in their respective references using MATLAB^©^ R2023a. The chemical kinetics of the two simulated Ni/ZrO_2_ catalysts were taken from [16], in which they were prepared by adopting two different methods: one was obtained using dielectric barrier discharge (DBD) plasma, and the other by calcination. In turn, the kinetics of the Ni/Al_2_O_3_ commercial catalyst were taken from [1]. The present study established CO_2_ conversion, CH_4_ selectivity, and H_2_ conversion in CO_2_ methanation depending on the catalyst preparation method, catalyst support, raw material composition, and gasification process conditions. 

According to the results, Ni/ZrO_2_-P had a better catalytic behavior than Ni/ZrO_2_-C, which corroborates that the catalyst preparation method influences catalyst activity. Also, the catalyst support affected CO_2_ conversion and H_2_ conversion. ZrO_2_-supported catalysts showed better CO_2_ conversion, but the Ni/Al_2_O_3_ commercial catalyst had a higher H_2_ conversion. However, the Ni/ZrO_2_p catalyst presented a better H_2_ conversion than the commercial Ni/Al_2_O_3_ at 700 °C and both S/B ratios.

Regarding the syngas composition, Ni/Al_2_O_3_ was more active for CO_2_ conversion and selective for CH_4_ when an S/B ratio of 1.0 for both temperatures was employed. In contrast, the H_2_ conversion was better for an S/B of 0.5.

Finally, the model presented in this study has the potential to be applied to evaluate the CO_2_ methanation process using different types of syngas derived from various biomass sources and operating conditions during the gasification process. By utilizing the same catalysts studied in this paper, researchers can conduct theoretical exploratory research to quickly estimate the impact of different variables on CO_2_ conversion, H_2_ conversion, and CH_4_ selectivity. This approach offers the advantage of saving both time and resources by providing a valuable method to identify the variables in the gasification process that promote favorable CO_2_ conversion, CH_4_ selectivity, and H_2_ conversion.

## Figures and Tables

**Table 1:** Kinetic parameters of Ni/ZrO_2_ catalysts [16].

Catalyst	E_a_ (kJ mol^−1^)	α	β	A (L g^−1^ h^−1^)
Ni/ZrO_2_-P	93.61	0.65	0.29	2.48 × 10^10^
Ni/ZrO_2_-C	93.12	0.44	0.54	6.93 × 10^9^

**Table 2:** Adsorption parameters of Ni/Al_2_O_3_ catalyst [1].

Parameter	K_CO_	K_H2O_	K_CO2_	K_H2_
Q (KJ/mol)	40.6	14.5	9.72	52.0
Ko (bar^−1^)	2.39 × 10^−3^	6.09 × 10^−1^	1.07	5.2 × 10^−5^

**Table 3:** Kinetic parameters of Ni/Al_2_O_3_ catalyst [1].

Parameter	k_CO2,meth_	k_RWGS_	k_CO,meth_
Ea (KJ/mol)	110	97.1	97.3
ko (mol/min·g)	1.14 × 10^8^	1.78 × 10^6^	2.23 × 10^8^

**Table 4:** Coffee pulp elemental analysis (%*w*/*w*).

N	C	H	O	S
2.53%	44.07%	5.93%	47.47%	0.00%

**Table 5:** Composition of syngas (%*v*/*v*) obtained from coffee pulp waste at different temperatures and steam/biomass (S/B) ratios.

Steam/Biomass (S/B) Ratio	S/B 0.5		S/B 1.0	
Temperature (°C)	700	800	700	800
Hydrogen (%)	48.46	46.51	54.41	50.19
Methane (%)	0.27	0.23	0.25	0.09
Carbon monoxide (%)	24.21	44.06	18.87	37.81
Carbon dioxide (%)	25.05	7.68	24.81	11.39
Ethane (%)	0.14	0.02	0.07	0.03
Ethylene (%)	1.03	0.80	0.59	0.09
Propane (%)	0.74	0.39	0.82	0.37
H_2_S (%)	0.1	0.31	0.18	0.03
H_2_/CO_2_ ratio	1.93	6.06	2.19	4.41

## Data Availability

The data are available upon request.

## References

[1] Champon I., Bengaouer A., Chaise A., Thomas S., Roger A.-C. (2019). Carbon dioxide methanation kinetic model on a commercial Ni/Al_2_O_3_ catalyst. J. CO2 Util..

[2] Scharl V., Fischer F., Herrmann S., Fendt S., Spliethoff H. (2020). Applying Reaction Kinetics to Pseudohomogeneous Methanation Modeling in Fixed-Bed Reactors. Chem. Eng. Technol..

[3] Brooks K.P., Hu J., Zhu H., Kee R.J. (2007). Methanation of carbon dioxide by hydrogen reduction using the Sabatier process in microchannel reactors. Chem. Eng. Sci..

[4] Shen L., Xu J., Zhu M., Han Y.-F. (2020). Essential role of the support for nickel-based CO_2_ methanation catalysts. ACS Catal..

[5] Romero-Sáez M., Dongil A., Benito N., Espinoza-González R., Escalona N., Gracia F. (2018). CO_2_ methanation over nickel-ZrO_2_ catalyst supported on carbon nanotubes: A comparison between two impregnation strategies. Appl. Catal. B Environ..

[6] Marchi M., Neri E., Pulselli F.M., Bastianoni S. (2018). CO_2_ recovery from wine production: Possible implications on the carbon balance at territorial level. J. CO2 Util..

[7] Clark J.H. (2007). Green chemistry for the second generation biorefinery—Sustainable chemica manufacturing based on biomass. J. Chem. Technol. Biotechnol..

[8] Aristizabal C., Alvarado P., Vargas A. (2020). Biorefinery concept applied to phytochemical extraction and bio-syngas production using agro-industrial waste biomass: A review. Ing. Investig..

[9] Esquivel P., Jiménez V.M. (2012). Functional properties of coffee and coffee by-products. Food Res. Int..

[10] Murthy P.S., Naidu M.M. (2012). Sustainable management of coffee industry by-products and value addition—A review. Resour. Conserv. Recycl..

[11] Aristizabal-Alzate C.E., Vargas-Ramírez A.F., Alvarado-Torres P.N. (2022). Simulation of methanol production from residual biomasses in a Cu/ZnO/Al_2_O_3_ packed bed reactor. Rev. Fac. Ing..

[12] Ussa P.A., Ocampo F., Kobl K., Louis B., Thibault-Starzyka F., Daturi M., Bazin P., Thomas S., Roger A.C. (2013). Catalytic CO_2_ valorization into CH_4_ on Ni-based ceria-zirconia. Reaction mechanism by operando IR spectroscopy. Catal. Today.

[13] Rönsch S., Köchermann J., Schneider J., Matthischke S. (2016). Global Reaction Kinetics of CO and CO_2_ Methanation for Dynamic Process Modeling. Chem. Eng. Technol..

[14] Ridzuan N.D.M., Shaharun M.S., Anawar M.A., Ud-Din I. (2022). Ni-Based Catalyst for Carbon Dioxide Methanation: A Review. Catalysts.

[15] Tongnan V., Ait-Lahcen Y., Wongsartsai C., Khajonvittayakul C., Siri-Nguan N., Laosiripojana N., Hartley U.W. (2021). Process intensification of methane production via catalytic hydrogenation in the presence of ni-ceo2/cr2o3 using a micro-channel reactor. Catalysts.

[16] Jia X., Zhang X., Rui N., Hu X., Liu C.-J. (2018). Structural effect of Ni/ZrO_2_ catalyst on CO_2_ methanation with enhanced activity. Appl. Catal. B Environ..

[17] Molino A., Chianese S., Musmarra D. (2016). Biomass gasification technology: The state of the art overview. J. Energy Chem..

[18] Ahmad A.A., Zawawi N.A., Kasim F.H., Inayat A., Khasri A. (2016). Assessing the gasification performance of biomass: A review on biomass gasification process conditions, optimization and economic evaluation. Renew. Sustain. Energy Rev..

[19] Bridgwater A. (1995). The technical and economic feasibility of biomass gasification for power generation. Fuel.

[20] Heidenreich S., Foscolo P.U. (2015). New concepts in biomass gasification. Prog. Energy Combust. Sci..

[21] Beohar H., Gupta B., Sethi V.K., Pandey M. (2012). Parametric Study of Fixed Bed Biomass Gasifier: A review. Int. J. Therm. Technol..

[22] Santos S.M., Assis A.C., Gomes L., Nobre C., Brito P. (2022). Waste Gasification Technologies: A Brief Overview. Waste.

[23] La Villetta M., Costa M., Massarotti N. (2017). Modelling approaches to biomass gasification: A review with emphasis on the stoichiometric method. Renew. Sustain. Energy Rev..

[24] Couto N., Rouboa A., Silva V., Monteiro E., Bouziane K. (2013). Influence of the biomass gasification processes on the final composition of syngas. Energy Procedia.

[25] da Silva J.C.G., Alves J.L.F., Mumbach G.D., Andersen S.L.F., Moreira R.d.F.P.M., Jose H.J. (2023). Hydrogen-rich syngas production from steam gasification of Brazilian agroindustrial wastes in fixed bed reactor: Kinetics, energy, and gas composition. Biomass-Convers. Biorefin..

[26] Sansaniwal S., Pal K., Rosen M., Tyagi S. (2017). Recent advances in the development of biomass gasification technology: A comprehensive review. Renew. Sustain. Energy Rev..

[27] Lin J., Ma C., Wang Q., Xu Y., Ma G., Wang J., Wang H., Dong C., Zhang C., Ding M. (2019). Enhanced low-temperature performance of CO_2_ methanation over mesoporous Ni/Al_2_O_3_-ZrO_2_ catalysts. Appl. Catal. B Environ..

[28] Cai M., Wen J., Chu W., Cheng X., Li Z. (2011). Methanation of carbon dioxide on Ni/ZrO_2_-Al_2_O_3_ catalysts: Effects of ZrO_2_ promoter and preparation method of novel ZrO_2_-Al_2_O_3_ carrier. J. Nat. Gas Chem..

[29] Grimalt-Alemany A., Skiadas I.V., Gavala H.N. (2018). Syngas biomethanation: State-of-the-art review and perspectives. Biofuels Bioprod. Biorefin..

[30] Knözinger H., Kochloefl K. (2003). Heterogeneous Catalysis and Solid Catalysts. Ullmann’s Encycl. Ind. Chem..

[31] Acar M.C., Böke Y.E. (2019). Simulation of biomass gasification in a BFBG using chemical equilibrium model and restricted chemical equilibrium method. Biomass-Bioenergy.

[32] Huang H.-J., Yuan X.-Z., Zhu H.-N., Li H., Liu Y., Wang X.-L., Zeng G.-M. (2013). Comparative studies of thermochemical liquefaction characteristics of microalgae, lignocellulosic biomass and sewage sludge. Energy.

[33] Fogler H.S. (2001). Elementos de Ingeniería de las Reacciones Químicas.

[34] Levenspiel O. (2002). Ingenieria de las reacciones químicas. J. Chem. Inf. Model..

